# Degree of hydrolysis, functional and antioxidant properties of protein hydrolysates from Grass Turtle (*Chinemys reevesii*) as influenced by enzymatic hydrolysis conditions

**DOI:** 10.1002/fsn3.1903

**Published:** 2021-06-24

**Authors:** Md. Serajul Islam, Wang Hongxin, Habtamu Admassu, Anwar Noman, Chaoyang Ma, Fu An wei

**Affiliations:** ^1^ State key Laboratory of Food Science and Technology Jiangnan University Wuxi Jiangsu province 214122 China; ^2^ National Engineering Research Center for Functional Food Jiangnan University Wuxi Jiangsu province 214122 China; ^3^ Department of Food Process Engineering, Biotechnology and Bioprocessing Center of Excellence Addis Ababa Science and Technology University Addis Ababa Ethiopia; ^4^ Department of Food Technology and Nutritional Science Mawlana Bhashani Science and Technology University Tangail Bangladesh; ^5^ Guangxi zhongtaikang Technology Industry Co., Ltd. Nanning Guangxi 530029 P. R. China

**Keywords:** Antioxidants activities, *Chinemys reevesii*, Enzymatic hydrolysis, Functional properties, Protein hydrolysate, Scanning electron microscopy (*SEM*)

## Abstract

Grass turtle muscle was hydrolyzed with papain enzyme to produce protein hydrolysate (PH) and the degree of hydrolysis (DH) was determined. Under optimal conditions, the highest DH was 19.52% and the yield was recorded as 17.26%. Protein content of the hydrolysates was ranged from 73.35% to 76.63%. Total amino acids were more than 96.77% for each PH. The PH obtained at DH 19.52% achieved excellent solubility and emulsifying activity which were 95.56% and 108.76 m^2^/g, respectively at pH 6. Foam capacity amounted 100% in PH of DH 19.52% at pH 2, and water‐holding capacity was 4.38 g/g. The antioxidant activity showed the strongest hydroxyl radical scavenging activity (95.25%), ABTS (84.88%), DPPH (75.89%), iron chelating (63.25%), and cupper chelating (66.90%) at DH 11.96%, whereas reducing power (0.88) at DH 19.52%. Thus, the findings indicated that utilization of grass turtle muscle protein hydrolysate is a potential alternative protein resource to improve the nutritional and functional properties in food ingredients and product formulations.

## Graphical abstract

This is the first study using enzymatic hydrolysis to prepare protein hydrolysates from Grass Turtle. Degree of hydrolysis was clearly affected by enzymatic hydrolysis conditions. Enzymatic hydrolysis process led to improve Grass Turtle protein hydrolysate properties. The highest protein solubility was 95.56% at pH 6 in protein hydrolysate obtained at DH 19.52%.Antioxidant activities were affected by molecular weights and amino acid profiles of protein hydrolysate.

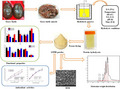

## 1 INTRODUCTION

Grass turtle (*Chinemys reevesii*) is an aquatic animal species of the Geoemydidae family, which is found in many countries, particularly in Hong Kong, China, Taiwan, Japan, and Korea (Dai et al., 2012). At present, research studies are focused on the practical utilization of various aquatic animal species products and their by‐products (Zou et al., 2017). Tortoises and turtles have long been used for foods and medicines in the East and Southeast Asia, while China is the largest consumer country in the world. The soft‐shelled turtles (*Pelodiscus sinensis*) is a commercially important and delicious aquatic species due to their higher nutritional value and medicinal benefits, where they can be used for anticancer, antioxidation, and reduces blood pressure. The global production of soft‐shelled turtles is estimated to be 355,000 tons in 2014 (Zhang et al., 2017). In addition, turtles have always been used as a tonic source in Chinese traditional medicine to make a person stronger, nourished, calmed, and also contains necessary elements as narrated in Materia Medica (Rawendra et al., 2014).

Enzymatic hydrolysis of food proteins is an effective way to reveal potent bioactive peptides (Rawendra et al., 2014). The functional properties and bioactivities of protein hydrolysates can be depend on molecular weight, hydrophobicity, and polar groups of the proteins which in turn are strongly affected by enzymatic hydrolysis conditions (Vilailak et al., 2007). Functional properties such as solubility, emulsification, foaming, and other properties of Protein hydrolysates are important in improving functional quality and bioavailability food products (Hall et al., 2018). Protein hydrolysates from natural resources produced through enzymatic hydrolysis possess various bioactivities. Among the bioactivities, antioxidant activity of protein hydrolysates is one of the focuses of current research. Antioxidants from a natural food products can demerit of potential health hazards of artificial antioxidants as food additives. Additionally, scientific information indicates that the consumption of natural antioxidants leads to reduce the risk of chronic diseases such as heart disease and cancer. PH with antioxidant activity in foods that plays an important role as a health protecting agent. Thus, applications of PH with antioxidant properties are more common in the food industry to improve the functional food (He et al., 2015).

As far as the author's knowledge is concerned, there are no information on the functional and antioxidant properties of protein hydrolysate from grass turtle muscles by using enzymatic hydrolysis in the utilization of food processing as a nutritional and functional value added products. Therefore, the aim of this study was to investigate the effects of enzymatic hydrolysis conditions on DH, functional and antioxidant properties of PH obtained from grass turtle muscles for the potential applications in food and pharmaceutical industries.

## 2 MATERIALS AND METHODS

### 2.1 Materials

#### 2.1.1 Samples

Grass turtle (*Chinemys reevesii*) is a kind of usual aquatic food in China. And the grass turtle we use comes from the breeding products of Guangxi zhongtaikang Technology Industry Co., Ltd., Nanning‐530029, Guangxi, P.R. China. Each grass turtle was rinsed thoroughly with tap water, and weighed, where their weights ranged between 1,135 and 1874 g and (length 21–25 × wide 12–17 cm) and then immediately slaughtered by using a knife. The muscles were separated from the other parts and by‐products were removed. Samples were put as fresh in ice box, then transported to laboratory. After consultation with relevant Chinese authorities, it is not an experimental animal, and it is unnecessary to issue animal ethics certificate. Finally, muscle(s) were homogenized, packed in vacuum plastic bags and stored at −20°C until further experiments. Before enzymatic hydrolysis process, the sample was transferred to the refrigerator at 4°C for 12 hr.

#### 2.1.2 Enzyme and Reagents

Papain enzyme (Enzyme activity 800u/mg, pH 5.5 ~ 6.5, 37 ~ 60 ℃ was purchased from Yuanye Bio‐Technology Co., Ltd. (Shanghai, China). The enzyme was directly stored at 4°C. All other chemicals and reagents used in the experiments were of high purity and analytical grade.

### 2.2 Methods

#### 2.2.1 Preparation of protein hydrolysates

**FIGURE 1:**
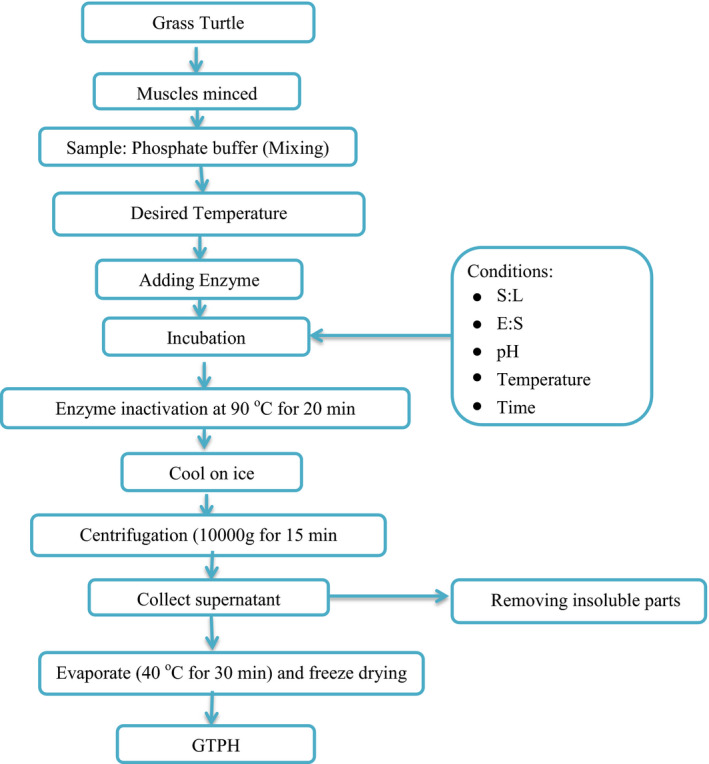
Scheme of the enzymatic hydrolysis process for the preparation of grass turtle muscles protein hydrolysates

Papain enzyme was used for enzymatic hydrolysis of grass turtle muscles. Single‐factor experiments (E:S, S:L, pH, temperature, and incubation time) were tested to obtain the optimal enzymatic hydrolysis conditions as shown in Table 1. Protein hydrolysates (PH) were prepared according to the method of Noman et al. (2018) with minor modifications. The $25\times 10^{‐3}M$ sodium phosphate buffer was used to keep the pH constant during the incubation time. The layout of the hydrolysis process is presented in Figure 1. The mixture was heated to 90°C for 20 min in a water bath (HH‐420, Wincom Company Ltd., Shanghai, China) to deactivate the further enzyme activity, immediately transferred to ice bath and centrifuged at 10000g at 4°C for 20 min (SCLOGEX‐ D3024R, Beijing, China). Finally, the supernatant was collected and concentrated by reducing the water content using vacuum evaporator (BC‐R203, Shanghai Biochemical Equipment Co., Ltd., China) at 40°C for 30 min, and then freeze‐dried (SCIENTZ‐10ND, Ningbo SCIENTZ Biotechnology Co., Ltd., Zhejiang, China) to find grass turtle protein hydrolysate (GTPH) powder, and stored at − 20°C for further analysis.

**Table 1:** The factors levels were used to obtain the optimum enzymatic hydrolysis conditions

Factors	Units	Symbol	Levels							
			1	2	3	4	5	6	7	8
Solid: Liquid	w/v	S:L	1:0.5	1:1	1:2	1:3	1:4	‐	‐	‐
Enzyme: Substrate	%	E:S	0.5	1	2	3	4	5	6	‐
pH	pH	pH	5	5.5	6	6.5	7	‐	‐	‐
Temperature	^o^C	T	35	40	45	50	55	60	65	70
Time	h	t	1	2	3	4	5	6	7	8

#### 2.2.2 Degree of hydrolysis determination

The degree of hydrolysis (DH) was investigated according to the modified method as described by Noman et al. (2018) with slight modifications. The volume of NaOH was used to calculate the amount of free amino groups. The total nitrogen was analyzed according to standard Kjeldahl method (AOAC, 1995). The percentage of free amino groups and the DH were calculated as follows.

$$\text{Free amino groups}\left(\%\right)=\frac{V\times C\times 0.014007}{W}\times 100$$

where, V (mL) is the volume of NaOH (0.1N) added, C is the concentration of the solution used for titration (0.1M NaOH), W (g) is the weight of sample.

#### 2.2.3 Yield determination

The yield of GTPH was determined according to a modified guideline (Dhanabalan et al., 2017), and calculated using the following equation:

$$\text{Yield}\left(\%\right)=\frac{\text{Weight of protein hydrolysate powder}\left(g\right)}{\text{Weight of raw material}\left(g\right)}\times 100$$

#### 2.2.4 Chemical composition analysis

Chemical composition (moisture, crude protein, lipid, and ash) were evaluated according to standard procedure (AOAC, 1995) with minor modifications. The moisture content of muscles was analyzed by an oven air drying at 105°C until a constant weight was obtained. The crude protein was estimated by using a standard micro Kjeldahl method (*N*% × 6.25). On the other hand, ash content was analyzed by incineration of the samples at 550°C in a muffle furnace until a constant weight. The total lipids were determined by using macro Soxhlet apparatus.

#### 2.2.5 Molecular weight distribution

Molecular weight (MW) profiles of muscle without enzyme hydrolysis and PH were investigated according to a guideline of W. Xu et al. (2017) with some modifications. Hundred (100 mg) of PH was taken into 15 ml glass tubes and diluted by 10 ml deionized water, the glass tubes were placed in an ultrasonic bath for 5 min and transferred into centrifuge tubes, centrifuged at 10,000 g for 10 min (SCLOGEX‐ D3024R, Beijing, China) at 4°C. Then supernatants were filtered and used for MW profile analysis by gel permeation chromatography using a HPLC system (Waters‐1525, USA). The TSK‐GEL 2000 SWXL (300 x 7.8 mm) column (Tosoh, Japan, Tokyo) was equilibrated with mobile phase composed of acetonitrile/water/trifluoroacetic acid 45/55/0.1（V/V).The column was eluted at a flow rate of 0.5 ml/min and monitored UV 220 nm and temperature at 30°C. Cytochrome C (12,384 Da), bacitracin (1,422 Da), Gly‐Gly‐Try‐Arg (GGYR) (451 Da), and Gly‐Gly‐Gly (GGG) (189 Da) were used as standards of MW.

#### 2.2.6 Amino acid analysis

Amino acids were analyzed by HPLC following an alkaline hydrolysis for tryptophan and other amino acids was performed by using the guideline of Al‐Farga et al. (2016) with some modifications. For tryptophan (total) analysis, 100 mg PH by 8 ml of 5 mol/L NaOH at 120°C for 22 hr under nitrogen gas and neutralized by 6.67 ml of 6 M HCl. On the other hand, other amino acids were determined, the same amount of sample was taken and hydrolyzed with 8 ml of 6 mol/L HCl under nitrogen gas and incubated in an oven at the same temperature and time, neutralized by 4.8 ml of 10M NaOH. For the free amino acids evaluation, PH taken 1,000 mg and diluted with 25 ml of 5% TCA then 1–2 hr stranded and centrifuged at 10,000 g for 10 min. Finally, 1 μl of solutions were injected into HPLC analytical column of 250 × 4.6 mm I.D, 5 μm particle size (Agilent Technologies, Palo Alto, California, USA). The determination was made by reverse‐phase high‐performance liquid chromatography (RP‐HPLC) (HP‐Agilent 1,100 model, Agilent Technologies) assembly system at 338 nm detection, 1.0 ml/min flow rate at 40°C column temperature. Mobile phase A was 7.35 mM/L C_2_H_3_NaO_2_/tri‐ethylamine/tetrahydrofuran (500:0.12:2.5, v/v/v) and adjusted to pH 7.20 ± 0.05 with CH_3_COOH while mobile phase B (pH 7.20 ± 0.05) was 7.35 mM/L C_2_H_3_NaO_2_/ CH_3_OH/acetonitrile (1:2: 2, v/v/v). The results were expressed as g/100g.

#### 2.2.7 Scanning electron microscopy (*SEM*)

The images of microstructure of muscles (dry powder) before enzymatic hydrolysis and PH powder were carried out by using a scanning electron microscope (Quanta 200, Fei Company, Netherlands) at an accelerating voltage of 5.0 kV and objective aperture 500 μm. All samples were coated by using a gold coater (Emitech K550X, Quorum Technologies Inc., UK), and the samples were examined at 160 × magnification.

#### 2.2.8 Color measurements

The color of the hydrolysate powders was measured using the Hunter Lab colorimeter (D65, UltraScan PRO, Shanghai, China). L*, a*, and b* parameters indicate white 100/ black 0, red positive/ green negative, yellow positive/blue negative, respectively of freeze‐dried PH. The color was evaluated as described by Thiansilakul et al. (2007).

#### 2.2.9 Water activity

Water activity (a_w_) measurements were performed using a Lab Master‐a_w_ (Novasina, Switzerland) with an accuracy of 0.001 at 25°C. After the calibration, the PH powders were placed in a sample chamber and kept until equilibrium was reached. Each sample was carried out at list three times.

#### 2.2.10 Functional properties of GTPH

##### Protein solubility

The protein solubility of the GTPH was evaluated according to procedure of Jain and Anal (2016) with minor modification. Two hundred milligram GTPH samples were dissolved by 20 ml deionized water, the solution was adjusted to pH 2 to 10 by using 0.1M HCl or 0.1M NaOH. Then, the solutions were incubated at 30°C with stirring (Blue pard Yiheng Technical Co., Ltd, Shanghai, China) at 150 rpm for 30 min, and centrifuged at 10,000 g for 15 min (SCLOGEX‐ D3024R, Beijing, China). The protein content of supernatant was determined by using the Kjeldahl method (AOAC, 1995). Finally, the percentage of solubility was calculated by following the equation.

$$\%\text{Protein solubility}=\frac{\text{Protein content in supernatant}}{\text{Total protein content in sample}}\times 100$$

##### Emulsifying properties

The emulsifying activity index (EAI) and emulsifying stability index (ESI) of GTPH were estimated by guideline (Jamdar et al., 2010; Pachecoaguilar et al., 2008) with some modifications. 1% GTPH sample solution was adjusted to pH 2, 4, 6, 8, and 10, then mixed with 10 ml soybean oil and homogenized (MORGEC, MBL50, Shanghai, China) at 19,000 rpm for 1 min. After emulsion formation, 50 μL was taken from the bottom by pipette and diluted with 5 ml of 0.1% sodium dodecyl sulfate (SDS) solution (w/v) at 0 and 10 min after homogenization. The absorbance of the solutions was analyzed at 500 nm by using UV‐1800PC Spectrophotometer (Shanghai Mapada Instruments Co., Ltd., China).

EAI and ESI calculated as the following formula

$$\text{EAI}\left(m^{2}g^{‐1}\right)=\frac{2\times 2.303\times A_{500}}{\emptyset \times S}$$

$$\text{ESI}\left(\min\right)=\frac{Ab_{0}\times 10}{Ab_{0}‐Ab_{10}}$$

$$\text{ESI}\left(\%\right)=100‐\left[\frac{Ab_{0}‐Ab_{10}}{Ab_{0}}\right]\times 100$$

where, Ab_0_ and Ab_10_ is the absorbance at 500 nm at 0 min to 10 time, A_500_ = Absorption value at 500 nm, S = Weight of sample (g), ∅= Oil volume fraction (0.25).

##### Water‐ and oil‐holding capacity

Water‐holding capacity (WHC) and oil‐holding capacity (OHC) were estimated by using a procedure of Noman et al. (2018) with minor modification. Each PH (0.5 g) was dissolved into 10 ml dd‐water or 10 ml soybean oil in a centrifuge tube and dispersed by vortex mixer (XW‐80A, Zhejiang, China) for 60 s. The water and oil dispersion were allowed to stand for 7 hr and 20 min, respectively, at 25°C and centrifuged (SCLOGEX‐ D3024R, Beijing, China) at 5000g for 25 min at 4°C. To get WHC the supernatant was filtered by using a filter paper (Whatman No. 1) and calculated by different weight, while the free oil was taken to obtain OHC from the weight difference. The results were mentioned as g/g PH.

##### Foaming capacity and foam stability

The foaming capacity (FC) and foam stability (FS) were estimated according to a modified method of Yeom et al. (2010) with minor modifications. One gram PH powder was taken and dissolved in 100 ml distilled water and adjusted to pH 2–10 using either 0.1 N NaOH or HCl. The solutions were poured into a 250 ml volumetric cylinder and the foam was prepared by using a homogenizer (MORGEC, MBL50, Shanghai, China) at 16,000 rpm for 2 min. The volume of foam was recorded directly after homogenization. On the other hand, FS was examined by measuring the fall in the volume of foam after every 2 min until 10 min. FC and FS were calculated according to the following question.

$$\text{FC}=\frac{V_{a}‐V_{b}}{V_{b}}\times 100$$

$$\text{FS}=\frac{\;\text{foam volume}\left(\text{mL}\right)}{\text{Initial foam volume}\left(\text{mL}\right)}\times 100$$

where, V_a_ is the before whipping Volume (mL) and V_b_ is the after whipping Volume (mL).

#### 2.2.11 Antioxidant properties of GTPH

All antioxidant (except hydroxyl radical) properties of GTPH were evaluated and measured by using a SpectraMax M5 Microplate Readers (SoftMax® Pro 5 serial number: SMP500‐16295‐OBVN, Beijing, China) in 96‐well plates, while hydroxyl radical using Spectrophotometer. PH was suspended (mg/ml) with deionized water and mixed for 1 min by vortex mixer (XW‐80A, Zhejiang, China). The concentration of PH (mg/mL) providing 50% inhibition of ABTS, DPPH, hydroxyl radical, and metal chelating activity, IC_50_ were calculated from the graph plotted with antioxidant concentration.

##### ABTS free radical scavenging activity

2,2’‐azino‐bis (3‐ethylbenzothiazoline‐6‐sulfonic acid) radical scavenging activity was analyzed by procedure of Hall et al. (2018) with minor modifications. Briefly, ABTS^+^ stock solution was diluted with ethanol: water (1:1, v/v) mixture to prepare a working solution with an absorbance of 0.75–0.80 at 734 nm. PH powder dissolved in ultrapure water to obtain concentrations of 1‐5mg/ml and centrifuged (SCLOGEX‐ D3024R, Beijing, China) at 5,000 g for 10 min. A 10 μL supernatant was allowed to react with 200 μL ABTS working solution for 10 min in dark conditions at 25°C and the absorbance recorded at 734 nm. ABTS activity was calculated as the following formula.

$$\text{ABTS}\left(\%\right)=\frac{{\text{Abs}}_{\text{Blank}}‐{\text{Abs}}_{\text{Sample}}}{{\text{Abs}}_{\text{Blank}}}\times 100$$

where, Abs_Blank_ = absorbance of control without sample and Abs_Sample_ = absorbance of sample.

##### Scavenging activity of DPPH radical

1,1‐diphenyl‐2‐picrylhydrazyl (DPPH) assay was analyzed by a guideline of Serpen et al. (2012) with some modification. Briefly, the working solution was prepared by water: ethanol (50:50, v/v) and absorbance was 0.75–0.80 at 525 nm. GTHP powder was diluted at different concentration from 1 to 20 mg/ml and then centrifuged at 5,000 rpm for 10 min (SCLOGEX‐ D3024R, Beijing, China). Finally, A 50 μL supernatant was allowed to react with 200 μL DPPH working solution for 30 min in dark conditions at 25°C and the absorbance recorded at 525 nm. DPPH activity was calculated as shown below formula.

$$\text{DPPH}\left(\%\right)=\frac{{\text{Abs}}_{\text{blank}}‐{\text{Abs}}_{\text{sample}}}{{\text{Abs}}_{\text{blank}}}\times 100$$

where, Abs_blank_ = absorbance of control sample and Abs_sample_ = absorbance of sample.

##### Reducing power capacity

Reducing power of protein hydrolysates was investigated as the procedure (Wu et al., 2003) with slight modification. Two hundred microliter of PH at concentrations of 1, 5, 10, and 15 mg/ml were added to 200 µl of 1% C₆FeK₃N₆ ($200\times 10^{‐3}$mM phosphate buffer (pH 6.6)) and mixed rapidly by vortex mixer (XW‐80A, Zhejiang, China) and incubated at 50°C for 20 min. Afterward, 200 µl of 10% TCA was added to the mixtures, and centrifuged at 10,000*g* for 10 min (SCLOGEX‐ D3024R, Beijing, China). The supernatant (200 µl) was mixed with 200 µl of deionized water and added 40 µl of 0.1% FeCl_3_. The mixture was allowed to react for 10 min and absorbance was analyzed at 700 nm.

##### Hydroxyl radical scavenging activity

Hydroxyl radical scavenging activity was evaluated according to the guideline of J. Wang et al. (2013) with some modifications. The mixtures were kept in water bath for 90 min at 25°C and the absorbance was analyzed at 522 nm by a UV‐1800PC Spectrophotometer (Shanghai Mapada Instruments Co., Ltd., China). The hydroxyl radical scavenging percentage was calculated by the following formula.

$$\text{Hydroxyl radical scavenging activity}\left(\%\right)=\left(1‐\frac{{\text{Ab}}_{s}‐{\text{Ab}}_{h}}{{\text{Ab}}_{c}}\right)\times 100$$

where, Ab_s_, Ab_h_, and Ab_c_ means the sample absorbance, H_2_O_2_ was substituted by distilled water, and sample was replaced by distilled water, respectively.

##### Fe (II) ion chelating activity

The Fe^2+^ chelating activity was evaluated as described (Hall et al., 2018; Naqash & Nazeer, 2013) with minor modification. Sample was prepared at the concentration of 1, 5, 10, 15, and 20 mg/ml. The absorbance was measured at 562 nm.

##### Copper (II) ion chelating activity

The Cu^2+^ chelating capability was assessed as described by Xu et al. (2014) with minor modifications. A 100 μl protein supernatant was mixed with 200 μl of 0.1mg/ml CuSO_4_ (50 mM sodium acted buffer pH 6.6) and mixed by vortex. After that 50 μL of 4 mM pyrocatechol violet solution added to react for 20 min at 25°C and the absorbance was recorded at 632 nm.

#### 2.2.12 Statistical Analysis

All experiments were performed at least in triplicate (*n* = 3). The results obtained were subjected to one‐way analysis of variance (ANOVA). Duncan's new Multiple Range Test was achieved to evaluate statistical significant difference between samples within the 95% confidence interval (*p* < .05) using IBM SPSS 22.0 software (SPSS Inc, Chicago, IL, USA).

## 3 RESULTS AND DISCUSSION

### 3.1 Optimization of enzymatic hydrolysis conditions

#### 3.1.1 Effect of enzyme‐to‐substrate ratio

**FIGURE 2:**
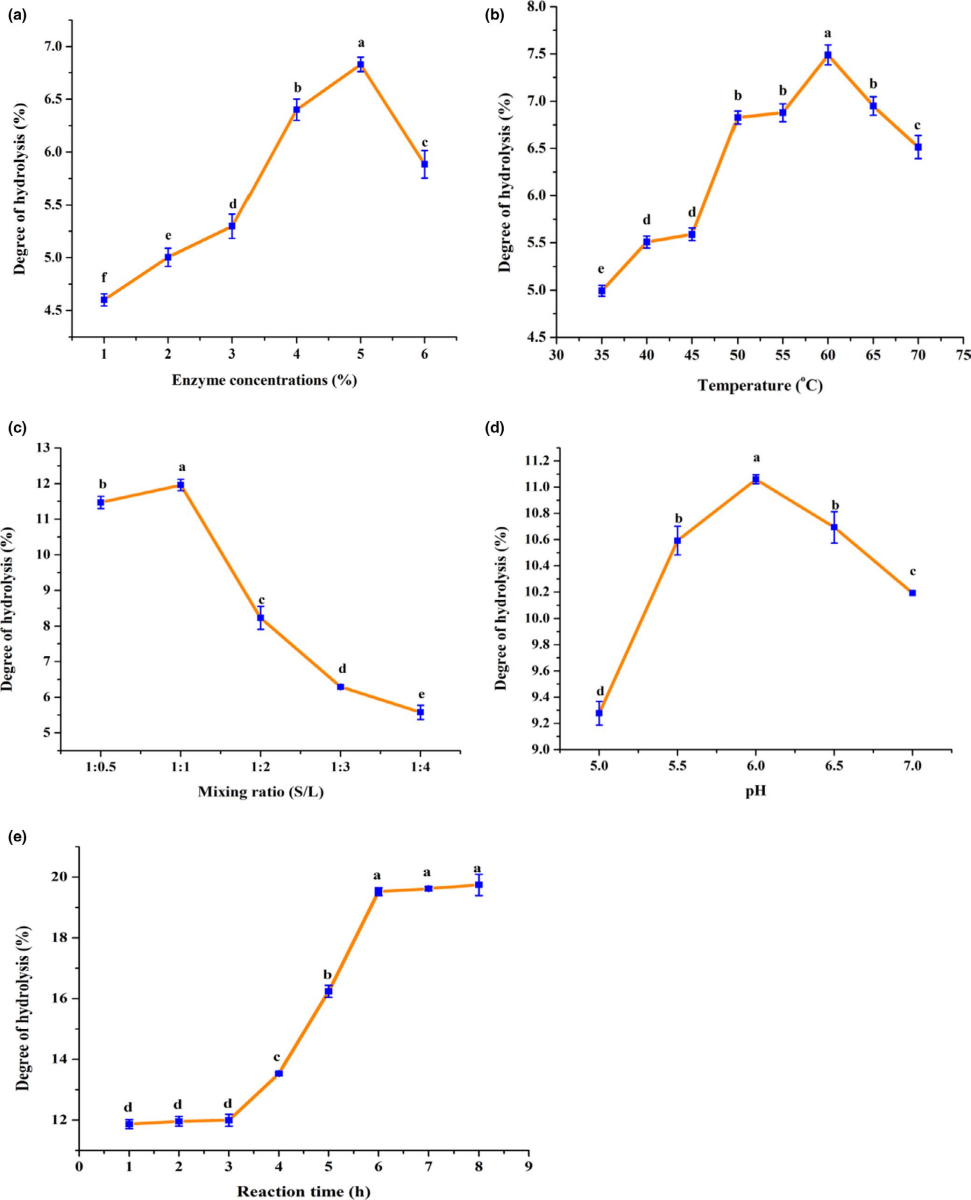
Effects of different conditions parameters on degree of hydrolysis, (a) Enzyme‐to‐substrate ratio, (b) Temperature, (c) Solid‐to‐liquid ratio (S/L), (d) pH, and (e) Time incubation. Data are expressed as mean ± S.D. of triplicate. Different small letters within each assay indicate significant differences (*p* < .05)

The effect of E:S ratio on the DH was evaluated within the six levels, and the results are revealed in Figure 2a. As it is observed, at 1% enzyme concentration, DH was 4.60% but when the enzyme concentration increased to 2%, the DH was 5.01%, and increased to 6.83% as the concentration of enzyme has increased to 5%. However, DH was decreased to 5.89% as the enzyme concentration is further increased to 6%, this probably due to enzymatic steric effect that prohibits in contact with the protein with catalytic sites enzymes which is promoting the enzymatic hydrolysis process, and the reduction of substrate diffusion, and saturation reaction rate (Noman et al., 2018). Thus, the optimum DH (6.83%) is obtained at 5% of enzyme concentration and the protein hydrolysate at this DH was chosen further experiment.

#### 3.1.2 Effect of temperature

The DH can be greatly affected by the hydrolysis temperature. The hydrolysis temperature was set at 35, 40, 45, 50, 55, 60, 65, and 70°C to evaluate its effect on the DH, and the results are presented in Figure 2b. When the reaction temperature increased from 35 to 60°C, the DH was significantly increased from 4.99% to 7.49%. However, by further increasing the reaction temperature to 65 and 70°C, a gradually decline in the DH was observed (Figure 2b). Such a reduction in the DH may be because of thermal denaturation of enzyme, hence leading to a decrease of DH (Noman et al., 2018). According to this result, temperature of 60°C was selected to further research, where this temperature was close to that was reported X. Wang and Zhang (2012) in their study on *Chlorella pyrenoidosa* by using papain enzyme.

#### 3.1.3 Effect of solid‐to‐liquid ratio

To obtain optimal solid‐to‐liquid ratio five levels (1:0.5 to 1:4, w/v) were used beside optimal enzyme‐to‐substrate ratio and temperature that was obtained above in this research, which are 5% and 60°C, respectively. The influence of solid‐to‐liquid mixing ratio on the DH has demonstrated in Figure 2c. The DH was 11.47% at ratio of 1:0.5, while DH significantly increased to 11.96% when the ratio of 1:1 was used. Further ratio increased to 1:2, 1:3, and 1:4 led to significantly decreased of DH. Therefore, Solid: Liquid ratio 1:1 was selected as optimal ratio to use in the next step. These finding is consistent with those found by Noman et al. (2018) who used the same mixing ratio for the optimization of enzymatic hydrolysis of Chinese sturgeon fish by using papain enzyme.

#### 3.1.4 Effect of pH

The effect of pH on the DH was investigated in the pH levels of 5, 5.5, 6, 6.5, and 7 as shown in Figure 2d. The DH was increased from 9.28% to 11.06% when the pH increased from 5 to 6. Nevertheless, by further increasing pH to 6.5 and 7, the DH decreased to 10.69% and 10.19%, respectively, such a decline in the DH probably attributed to the denaturation of protein structure of the enzyme (Noman et al., 2018). As a result, pH 6 was selected as optimal pH for further investigation. Similar results were obtained by Wang and Zhang (2012) who revealed that the optimum hydrolysis pH was 6 by using papain enzyme in *Chlorella pyrenoidosa*.

#### 3.1.5 Effect of time

The effect of reaction time on the DH was measured within the range of 1–8 hr and these results are shown in Figure 2e. It observed that, when the reaction time increased from 1 to 6 hr, the DH was significantly raised from 11.87% to 19.52%. Nonetheless, upon extending the incubation time 7 and 8 hr, showed no significant increase of DH obtained at 6 hr. According to these results, 6 hr was chosen as the optimal incubation time. Therefore, the optimization profile indicates that an optimum DH (19.52%) can be achieved with including an enzyme‐to‐substrate ratio of 5%, temperature 60°C, substrate to liquid ratio 1:1, pH 6, and incubation time 6 hr. DH under the optimal condition was highly compatible with those reported (Chalamaiah et al., 2010; Wang & Zhang, 2012) which were 17.1% and 14.33% from meriga egg and *Chlorella pyrenoidosa, respectively,* under optimal conditions.

### 3.2 Yield

The GTPH yields obtained in this study are demonstrated in Table 2. GTPH yield was found to be 17.26% of PH obtained at DH 19.52%, followed by 12.11% at DH 13.53%, while PH obtained at DH 11.96% was 10.43%. This yield percentage under optimal conditions (17.26%) supported by Noman et al. (2018) who obtained 17.47% yield from Chinese sturgeon by using the same enzyme. The variation of yield results depending on raw materials and enzymatic hydrolysis conditions such as incubation time which led to different DH in our study under optimal conditions.

**Table 2:** Yield, Chemical composition, color, a_w_, and functional properties of raw muscle and GTPH at various DH (*n* = 3, mean ± *SD*)

Parameters	Fresh sample	Hydrolyzed		
		DH 11.96% (2 hr)	DH 13.53% (4 hr)	DH 19.52% (6 hr)
Yield (%)	‐	10.43 ± 0.10^c^	12.11 ± 0.15^b^	17.26 ± 0.5^a^
Moisture (%)	74.76 ± 1.43	7.18 ± 0.09^a^	6.94 ± 0.11^ab^	6.79 ± 0.18^b^
Protein (%)	22.48 ± 0.48	73.35 ± 1.02^b^	74.71 ± 0.86^b^	76.63 ± 0.60^a^
Fat (%)	1.36 ± 0.03	0.22 ± 0.00^b^	0.39 ± 0.01^a^	0.14 ± 0.00^c^
Ash (%)	1.40 ± 0.01	8.15 ± 0.10^a^	7.53 ± 0.10^b^	7.57 ± 0.14^b^
Color				
L*	‐	85.82 ± 1.94^a^	81.59 ± 2.10^b^	79.34 ± 1.41^b^
a*	‐	1.19 ± 0.06^b^	1.15 ± 0.03^b^	1.76 ± 0.02^a^
b*	‐	16.74 ± 0.07^c^	17.88 ± 0.15^b^	19.97 ± 0.27^a^
Water activity	‐	0.21 ± 0.00^b^	0.24 ± 0.00^a^	0.19 ± 0.00^c^
Functional properties				
WHC (g/g PH)	‐	3.99 ± 0.00^b^	4.38 ± 0.01^a^	2.87 ± 0.19^c^
OHC (g/g PH)	‐	2.98 ± 0.04^b^	2.33 ± 0.07^c^	3.66 ± 0.05^a^

Note

Means with different letters in each row are significantly different (*p* < .05).

### 3.3 Chemical composition

The Chemical profiles of raw material and GTPH obtained by using papain enzyme hydrolysis are displayed in Table 2. Crude protein content of fresh muscles was 22.48%. Enzymatic hydrolysis process led to increase the protein content in protein hydrolysates which was the highest (76.63%) at the DH 19.52% (6 hr) followed by 74.71% of PH obtained at DH 13.53% (4 hr), while the PH gained at DH 11.96% (2 hr) contained the lowest value (73.35%), without significant differences (*p* < .05) between PH at DH 13.53% and 11.96%.

These results possibly be due to grass turtle (*Chinemys reevesii*) muscle may contain higher soluble and hydrolysable protein, in addition, the insoluble undigested removal during the centrifuge process (Thiansilakul et al., 2007). Thus, these GTPH could be an essential source of protein. On the other hand, fat content in the GTPH obtained at different DH ranged from 0.14% to 0.39%, these results were lower than (Vilailak et al., 2007) who found 0.67%. Finally, ash content in PH obtained at DH of 11.96% was significantly higher (8.15%) than PH found at DH 13.53% and DH 19.52% which were 7.53% and 7.57%, respectively. Ash content of GTPH was agreement with Chinese sturgeon muscle reported by Noman et al. (2018). Differences of proximate composition of PH may be due to the difference in raw materials and enzymatic hydrolysis conditions applied.

### 3.4 Molecular weight (MW) profile

**FIGURE 3:**
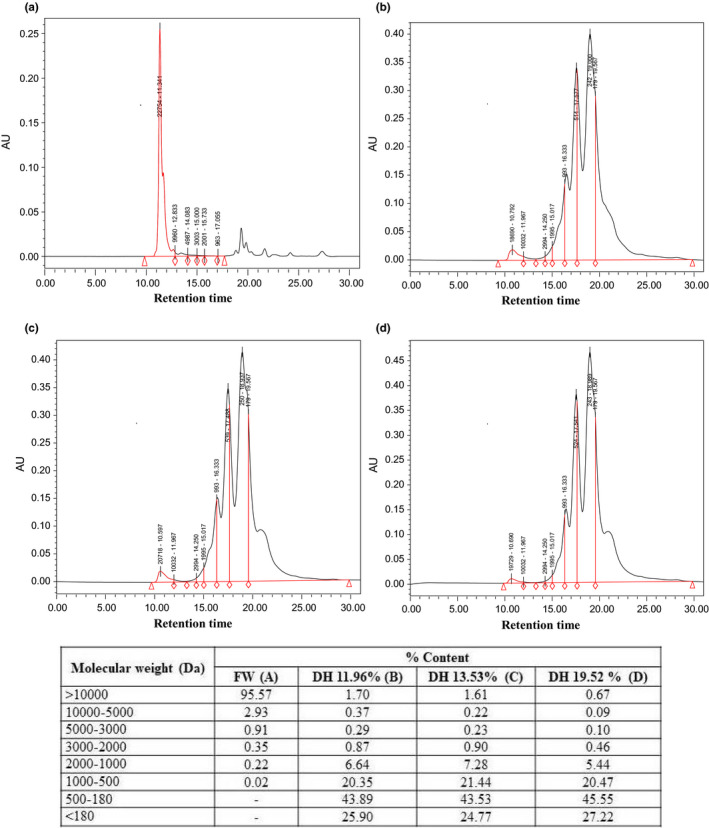
Molecular weight distribution of raw and GTPH by using papain enzyme under various degree of hydrolysis: (a) FW (fresh weight), (b) DH 11.96% (2 hr), (c) DH 13.53% (4 hr), and (d) DH 19.52% (6 hr)

The MW distribution of nonhydrolyzed sample and PH obtained from grass turtle muscles under the various DH are demonstrated in Figure 3. The results showed that, the PH obtained at the various DH have small molecular mass peptides compared with untreated sample where higher MW was > 95%, probably due to the effect of enzymatic hydrolysis which led to breakdown of peptides bounds. The MW ≤ 1,000 Da fractions were more than 89% during various hydrolysis periods. The main proportion of GTPH peptides ranged between 180 and 500 Da, which were 43.53 to 45.55% from total MW. These result is closely associated with the finding of Wasswa et al. (2007) who reported that proportion of the low MW peptides increased when the DH increased. The dietary proteins rich in low MW peptides could be more available in the food system and may highly contributing to nutritional value (Noman et al., 2018). Therefore, PH could be used in food products to improve the nutritional value.

### 3.5 Amino acid composition

Amino acid composition affects the nutritional value of PH particularly essential amino acids beside their effects on the functional properties (Santos et al., 2011). Nineteen different amino acids (total and free) were investigated, which are displayed in Table 3. Inthe fresh muscles, the total amino acids were 21.45 g/100g fresh sample and the major amino acids content such as glutamic acid, aspartic acid, and lysine were 4.03, 2.26, and 1.94 g/100g in fresh sample, respectively. These results were excellently higher than reported by Liang et al. (2018) who studied on Chinese soft‐shelled turtle muscle (77.01–84.44 mg/100 mg dry weight). When samples subjected to enzymatic hydrolysis process, the PH obtained at DH 19.52% and DH 11.96% were achieved the highest total amino acids with no significant difference (*p* < .05) which were 98.95 and 99.46 g/100g protein, respectively, while the PH obtained at DH of 13.53% was significantly lowest value 96.77 g/100g protein. In our results, total amino acid was higher than that obtained by Ovissipour et al. (2009); Dong et al. (2020); and dos Santos et al. (2011) from Persian sturgeon viscera; Cordyceps militaris chicken soup; and Bluewing Searobin by using enzymes, respectively. The major amino acids in GTPH were glutamic, aspartic, and lysine which ranged 17.49–17.66, 9.80–10.92, and 8.42–9.25 g/100g protein, respectively. Aspartic acid and glutamic acid are the most important amino acids that contribute to palatability. In addition, alanine, glycine, serine, and threonine deliberate taste sweet (Sriket et al., 2007).

**Table 3:** Amino acid composition of raw muscle (s) and GTPH obtained by using papain enzyme (*n* = 3, mean ± *SD*)

Amino acids	Muscles (g/100 g sample)		Protein Hydrolysate (g/100 g protein)					
			DH 11.96% (2 hr)		DH 13.53% (4 hr)		DH 19.52% (6 hr)	
	TAA	FAA*	TAA	FAA	TAA	FAA	TAA	FAA
Isoleucine	1.18 ± 0.03	0.01	4.59 ± 0.12^a^	0.23 ± 0.01^b^	4.43 ± 0.09^ab^	0.29 ± 0.01^a^	4.25 ± 0.12^b^	0.30 ± 0.01^a^
Leucine	1.75 ± 0.02	0.01	7.27 ± 0.17^a^	0.46 ± 0.02^b^	7.06 ± 0.18^ab^	0.59 ± 0.02^b^	6.77 ± 0.18^b^	1.68 ± 0.27^a^
Lysine	1.94 ± 0.05	0.01	8.91 ± 0.23^a^	0.50 ± 0.03^c^	9.25 ± 0.31^a^	0.64 ± 0.04^b^	8.42 ± 0.16^b^	1.27 ± 0.07^a^
Methionine	0.39 ± 0.01	0.00	2.40 ± 0.06^a^	0.15 ± 0.01^c^	2.37 ± 0.04^a^	0.18 ± 0.01^b^	2.24 ± 0.06^b^	0.86 ± 0.02^a^
Phenylalanine	0.92 ± 0.01	0.01	3.64 ± 0.11^ab^	0.21 ± 0.01^c^	3.49 ± 0.07^b^	0.26 ± 0.01^b^	3.70 ± 0.09a	0.58 ± 0.00^a^
Histidine	0.68 ± 0.02	0.04	3.12 ± 0.07^a^	0.06 ± 0.00^c^	2.82 ± 0.19^b^	0.07 ± 0.00^b^	2.84 ± 0.09^b^	0.39 ± 0.01^a^
Threonine	0.89 ± 0.02	0.02	3.87 ± 0.13^a^	0.01 ± 0.00^b^	3.57 ± 0.14^b^	0.01 ± 0.00^b^	3.54 ± 0.14^b^	0.35 ± 0.01^a^
Tryptophan	0.09 ± 0.00	0.00	2.84 ± 0.06^a^	0.08 ± 0.00^b^	2.59 ± 0.11^b^	0.10 ± 0.01^a^	2.55 ± 0.05^b^	0.11 ± 0.01^a^
Valine	1.17 ± 0.05	0.02	5.00 ± 0.28^a^	0.28 ± 0.01^b^	4.87 ± 0.2^1a^	0.34 ± 0.02^a^	4.67 ± 0.13^a^	0.22 ± 0.01^c^
**ΣEAA**	**9.00 ± 0.23**	**0.12**	**41.63 ± 1.19**	**1.97 ± 0.09**	**40.46 ± 1.31**	**2.49 ± 0.12**	**38.99 ± 1.01**	**5.75 ± 0.40**
Arginine	1.42 ± 0.02	0.07	6.41 ± 0.19^b^	0.00 ± 0.00^b^	6.07 ± 0.26^b^	0.00 ± 0.00^b^	6.99 ± 0.24a	1.33 ± 0.03^a^
Proline	0.88 ± 0.03	0.04	4.38 ± 0.14^b^	0.28 ± 0.01^b^	4.28 ± 0.17^b^	0.38 ± 0.01^a^	5.05 ± 0.06a	0.04 ± 0.00^c^
Glycine	1.31 ± 0.06	0.02	7.15 ± 0.13^b^	0.58 ± 0.04^b^	6.62 ± 0.12^b^	0.73 ± 0.03^a^	6.81 ± 0.16a	0.40 ± 0.00^c^
Cystine	0.04 ± 0.00	0.00	0.33 ± 0.02^a^	0.01 ± 0.00^b^	0.31 ± 0.01^a^	0.01 ± 0.00^b^	0.18 ± 0.01b	0.14 ± 0.01^a^
Tyrosine	0.59 ± 0.01	0.02	2.80 ± 0.12^a^	0.21 ± 0.01^c^	2.72 ± 0.08^a^	0.27 ± 0.01^b^	2.86 ± 0.08a	0.51 ± 0.00^a^
Taurine	0.11 ± 0.00	0.05	0.63 ± 0.04^a^	0.04 ± 0.00^c^	0.65 ± 0.02^a^	0.06 ± 0.00^b^	0.62 ± 0.02a	0.19 ± 0.01^a^
**ΣCEAA**	**4.35 ± 0.13**	**0.20**	**21.71 ± 0.62**	**1.13 ± 0.06**	**20.66 ± 0.65**	**1.44 ± 0.06**	**22.50 ± 0.56**	**2.62 ± 0.05**
Alanine	1.26 ± 0.02	0.03	5.97 ± 0.17^a^	0.63 ± 0.02^c^	5.86 ± 0.24^a^	0.80 ± 0.02^a^	5.67 ± 0.14^a^	0.67 ± 0.00^b^
Aspartic acid	2.26 ± 0.07	0.02	9.86 ± 0.37^b^	0.51 ± 0.01^b^	9.80 ± 0.16^b^	0.64 ± 0.02^a^	10.92 ± 0.39^a^	0.34 ± 0.02^c^
Glutamic acid	4.03 ± 0.14	0.08	17.66 ± 0.42^a^	1.14 ± 0.04^c^	17.50 ± 0.54^a^	1.46 ± 0.06^a^	17.49 ± 0.52^a^	1.23 ± 0.00^b^
Serine	0.56 ± 0.03	0.02	2.64 ± 0.01^b^	0.03 ± 0.00^b^	2.48 ± 0.02^c^	0.04 ± 0.00^b^	3.38 ± 0.11^a^	0.34 ± 0.01^a^
**ΣNEAA**	**8.10 ± 0.27**	**0.14**	**36.13 ± 0.96**	**2.32 ± 0.07**	**35.65 ± 0.95**	**2.94 ± 0.11**	**37.46 ± 1.15**	**2.58 ± 0.03**
**Total**	**21.45 ± 0.62**	**0.46**	**99.46 ± 2.77**	**5.41 ± 0.23**	**96.77 ± 2.90**	**6.87 ± 0.28**	**98.95 ± 2.71**	**10.95 ± 0.48**

Abbreviations: CEAA:Conditionally essential amino acid; EAA: essential amino acids; NEAA, Nonessential amino acids;TAA:Total amino acid.

* Standard deviation of FAA results was 0.00 except glutamic acid was 0.01.

On the other hand, free amino acids increased from 0.46 g/100 g fresh sample to 5.41, 6.87, and 10.95 g/100 g protein in PHs obtained at DH of 11.96%, 13.53%, and 19.52%, respectively. These differences between free amino acids content in the fresh sample and PH possibly due to effect of enzymatic hydrolysis process. Wu et al. (2003) reported that most of the free amino acids increased after enzymatic hydrolysis.

### 3.6 Scanning electron microscopy

**FIGURE 4:**
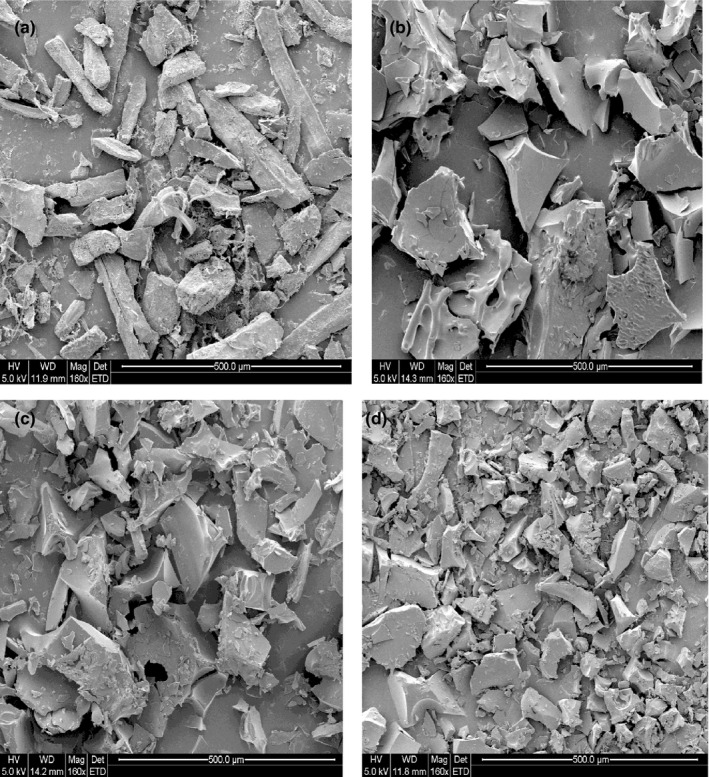
Scanning electron microscopy (*SEM*) of (a) before enzyme hydrolysis, (b) PH of DH 11.96%, (c) PH of DH 13.53%, and (d) PH of DH 19.52% from grass turtle muscles ((Mag = 160×; AV = 5.0 kV)

*SEM* images of fresh sample (dry) and PH for a DH of 11.96%, 13.53%, and 19.52% are presented in Figure 4. From SEMimages, it has observed that the protein has degraded into small fragments after enzyme hydrolysis, which led to reduction in particle size of the GTPH (Figure 4b,c, and d) compared with untreated sample of grass turtle muscles (Figure 4a) under the same SEMparameters (Mag = 160×; AV = 5.0 kV). These findings are closely associated of (Agrawal et al., 2019; Elavarasan & Shamasundar, 2016) who mentioned that protein has degraded into small fragments and particle size reduction after treatment by enzyme. Bao et al. (2017) reported that reduced the particle size may attributed to the high solubility.

### 3.7 Color assessment

The color of freeze‐dried PH evaluated by colorimeter is shown in Table 2. As a result, L* (lightness) value was significantly higher (85.82) at DH 11.96% followed by PH obtained 81.59 and 79.34 obtained under the DH 13.53% and DH 19.52%, respectively. Analyzing the values of a* and b*, the PH tended to redness and yellowness. These values were increased when DH increased in the range of a*=1.15–1.76 and b*=16.74–19.97, correspondingly. In our study L*, and a* value was higher than reported by Thiansilakul et al. (2007) who found brownish yellow color (L* = 58.00, a* = 8.38, b* = 28.32) of round scad protein hydrolysate powder. Color differences may be attributed due to the effect of enzymatic hydrolysis on the sample. Several studies have shown that the varying color is mainly depended on the presence of pigments in the muscle, in addition to hydrolysis conditions and nature of raw materials (Rodrigues Freitas et al., 2016; Thiansilakul et al., 2007). Overall, L* value was significantly (*p* < .05) decreased when the DH was increased, while, a* and b* were gradually increased.

### 3.8 Water activity

Water activity of a food determines its stability, which is based on availability of water and molecular mobility, and characterizes mainly the physico‐chemical and biological degradation in foods (Roudaut et al., 2004). The experimental results of a_w_ are presented in Table 2. The results observed that the best value was 0.19 in PH at DH 19.52% followed by 0.21 at DH 11.96%, while the highest value was 0.24 at DH 13.53%. In these GTPH obtained lower water activity, which could be contributing to excellent stability and increase storage life. Where the water activity is in range of 0.54–0.64 leads to increasing the hardness of food products, and maillard reaction occurs when the a_w_ is in the range of 0.74 to 0.84, as well as encourages the growth of some microorganisms (Rao et al., 2016).

### 3.9 Functional properties of GTPH

#### 3.9.1 Protein Solubility

**FIGURE 5:**
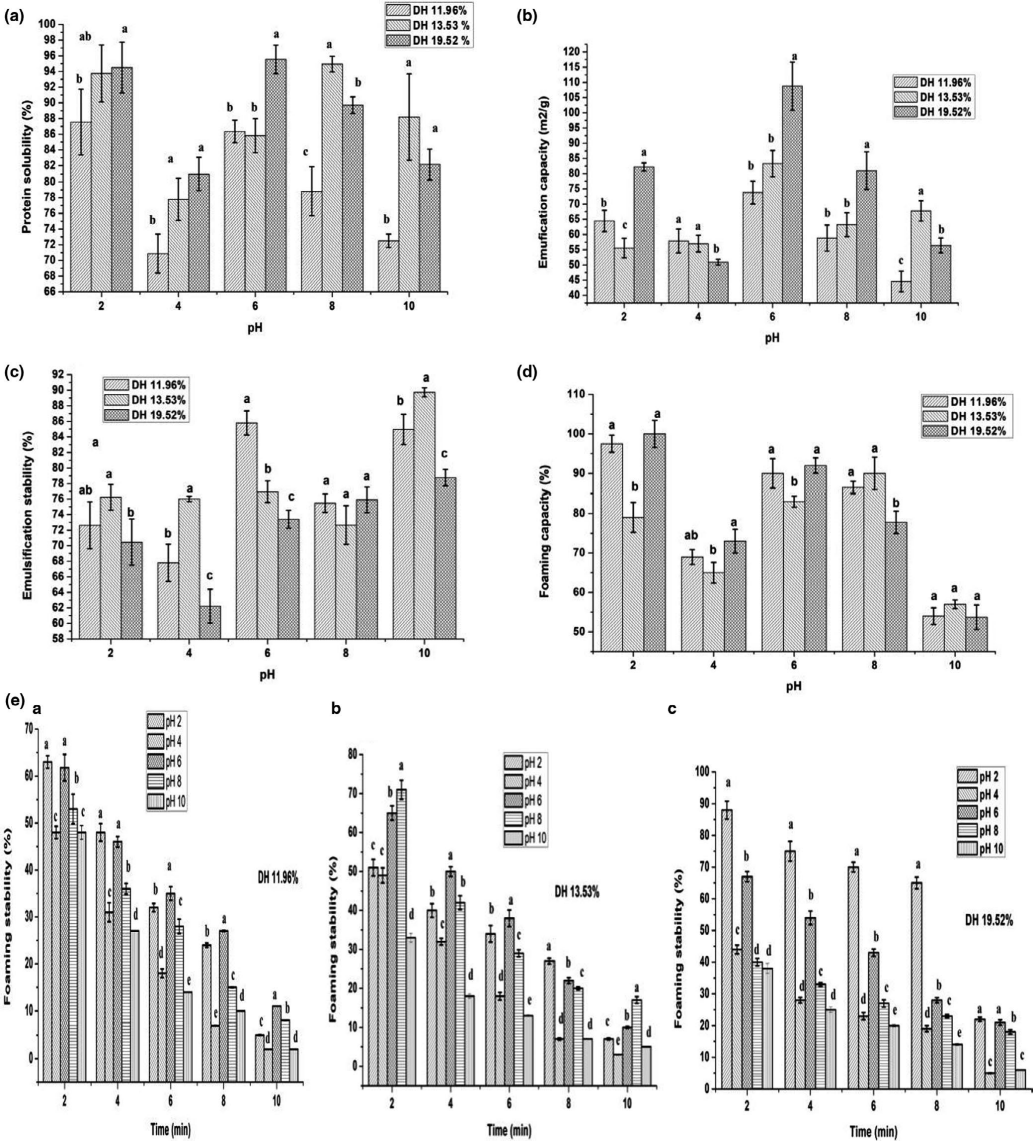
Functional properties of GTPH including (a) Protein solubility (%), (b) Emulsifying activity index (m^2^/g), (c) Emulsification stability index (%), (d) Foaming capacity (%), and (E.a, E.b, E.c) Foaming stability (%). The values represent means of three independent experiment ± *SD*. Different characters indicates significant differences at the each pH level (*p* < .05)

Solubility is an importance functional property of protein hydrolysates, which is required in food industries to controls the utilization of the product in many applications such as gels, emulsions, and foams (Thiansilakul et al., 2007). The protein solubility of GTPH with different DH in the pH ranges of 2–10 are presented in Figure 5a. All the protein hydrolysates showed higher solubility (70.87%–95.56%) depending on the pH used. The highest solubility rate was observed at pH 6 (>95%) in PH at DH 19.52%, while the lowest value was obtained at pH 4 (70.87%) in PH obtained at DH 11.96%. These results may be attributed due to DH and small molecular weights of peptides < 1,000 Da, this findings supported by Naqash and Nazeer (2013) who reported that degradation of proteins to smaller peptides led higher solubility. In this study, the solubility of GTPH was quite low at pH 4, which was in agreement of Vilailak et al. (2007). Similar results were reported by Foh et al. (2010) who mentioned the isoelectric points (pI) of protein are between pH 4.5 and 5.5, and also near to this range, at which the net charge of the original proteins are minimized, and thus more protein and protein interaction, and less protein–water interaction occur. As a result, the protein solubility is decreased, whenever, the pH moves away from this point, increases protein and water interactions. These results suggest that GTPHs showed excellent protein solubility, which may providing to attractive appearance and smooth mouth feel of the food products (Thiansilakul et al., 2007).

#### 3.9.2 Emulsifying properties

EAI and ESI of hydrolysates from GTPH with various DH (11.96%, 13.53%, and 19.52%) are revealed in Figure 5b and c. The result shows that EAI and ESI of both are significantly affected by pH level, where EAI was the highest capacity 108.76 m^2^/g in PH of DH 19.52% at pH 6 followed by 83.31 m^2^/g in PH of DH 13.53%. The lowest EAI found 44.53m^2^/g at pH 10 in PH of DH 11.96%. These occurred may be due to protein solubility, DH, and small molecular weight peptides. Santos et al. (2011) reported that emulsifying properties are mostly influenced by protein solubility, DH, and molecular size from Bluewing Searobin by using microbial enzymes.

On the other hand, ESI values were significantly different (*p* < .05) depending on the DH and pH levels used except pH 8 as shown in Figure 5c. It's observed that the maximum emulsions stability was 89.73% in PH obtained under DH 13.53% at pH 10, while the minimum was 62.23% at pH 4 of DH 19.52%. Generally, ESI was high in pH 10 and lowest at pH 4, which probably be due to the isoelectric point at pH 4 (Naqash & Nazeer, 2013). These results may have the relationship with hydrophobic amino acids such as isoleucine, leucine, valine, alanine, and glycine (Table 3). Vilailak et al. (2007) found that more hydrophobic peptides contribute to the stability of the emulsion.

#### 3.9.3 Water‐ and oil‐holding capacity

The WHCand OHCare affected by molecular weights. As shown in Table 2, the highest values of WHCwas obtained at DH 13.53% recorded as 4.38 gwater/g PH, followed by at DH 11.96% which was achieved 3.99 gwater/g PH, while the lowest value was 2.87 gwater/g PH at DH 19.52%. The WHCis affected by molecular weight size, where increasing the DH leads to the production of small molecular weight peptides, thus decreases the WHC(Santos et al., 2011). On the other hand, protein hydrolysate at DH 19.52% was achieved the highest OHC(3.66 g oil/g PH), and protein hydrolysate of DH 11.96% and DH 13.53% were found to be 2.98 and 2.33 goil/g PH, respectively. In this study, WHCand OHCvalues were higher than that reported by Noman et al. (2018) but lower than by S. He et al. (2016) who evaluated the OHC of rock lobster hydrolysate. Santos et al. (2011) found that OHCwas ranged from 3.86 to 5.12 and from 2.95 to 3.22 ml oil/g protein of Bluewing Searobin hydrolysates obtained at DH 15% and 10% by using Alcalase and Flavourzyme enzyme, respectively. In the same context, OHCis an important functional property, which influences the taste of products and necessary to application in meat products.

#### 3.9.4 Foaming capacity and stability

The foaming properties of GTPH with different DH are displayed in Figure 5d and Figure 5e (a, b and c). Figure 5d shows the highest FC was 100% at pH 2 in PH with DH 19.52% shadowed by PH of DH 11.96% (97.50%), while the PH of DH 13.53% was achieved the high FC (90%) at pH 8. All the PH was the lowest of FC at pH 4, these result is consistent with that was found by Noman et al. (2018) from Chinese sturgeon hydrolysate. On the other hand, the PH at DH 19.52% was achieved the highest foaming stability after 10 min, which significantly decreased over time from 88% at 2 min to 6% at 10 min (Figure 5e.c).

All protein hydrolysate are affected by pH values where the highest foaming stability at pH 6 and the lowest stability at pH 4, may be due to low protein solubility at isoelectric points in pH 4 (Naqash & Nazeer, 2013). On the other hand, Chalamaiah et al. (2010) mentioned that the foam capacity and stability increased with high protein content in meriga fish egg hydrolysates obtained by using Alcalase and papain enzymes. Generally, good foaming properties mainly depend on transportation, permeation and redisposition of molecules at the air/water interface (Vilailak et al., 2007).

### 3.10 Antioxidants properties

#### 3.10.1 ABTS free radical scavenging activity

**FIGURE 6:**
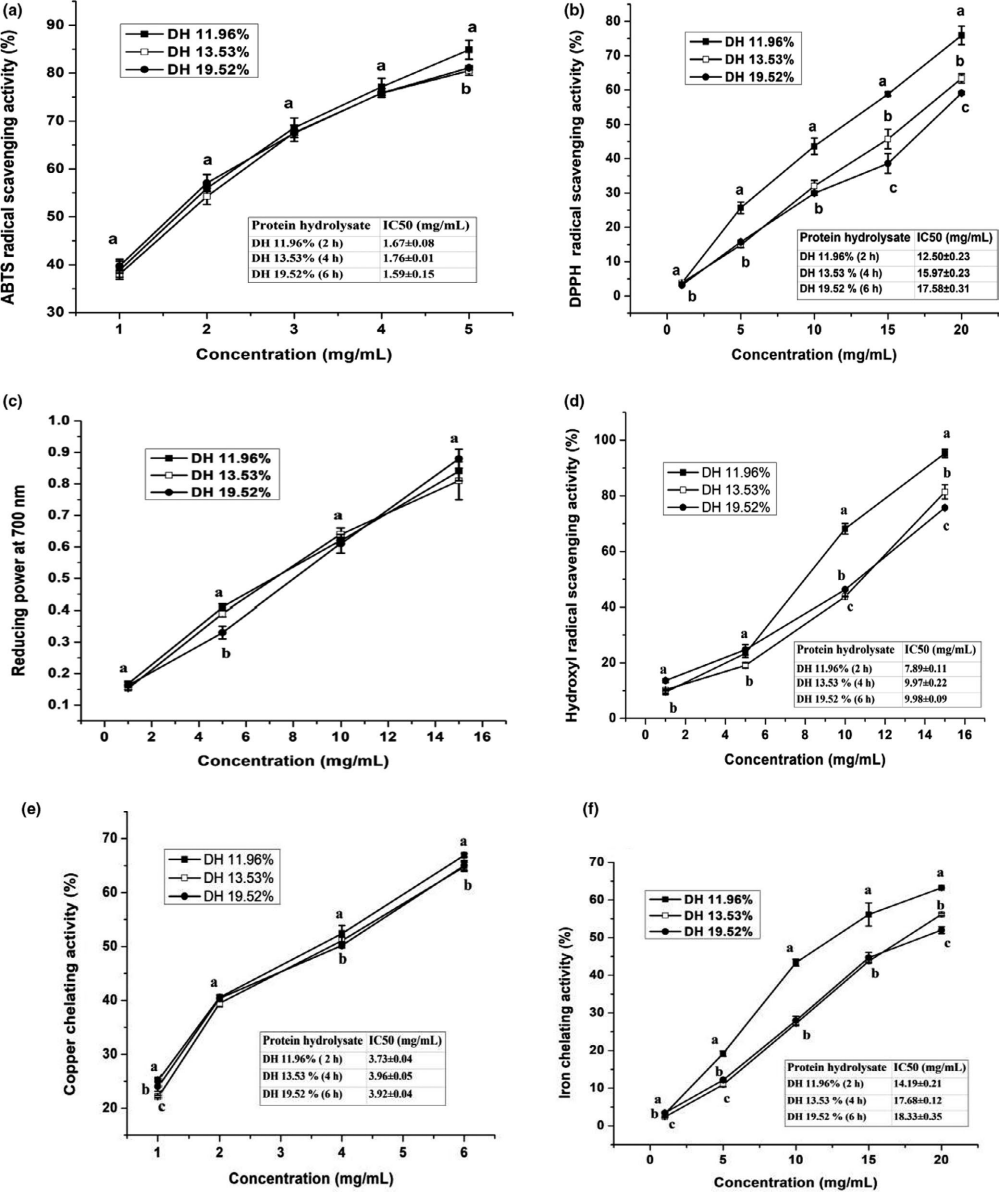
Comparison of antioxidant activities and IC_50_ of protein hydrolysates at different DH: (a) ABTS free radical scavenging activity (%), (b) DPPH free radical scavenging activity (%), (c) Reducing power capacity at 700 nm, (d) Hydroxyl radical scavenging activity (%), (e) Fe^2+^ chelating activity (%) as well as (f) Cu^2+^ chelating capacity (%). Values were presented as mean ± *SD*. Different small characters indicates significant differences (*p* < .05)

ABTS^•+^ scavenging activity of different PH at DH are revealed in Figure 6a. From these results, PH obtained at DH 11.96% (2 hr) was showing significantly higher ABTS activity (84.88%) followed by PH at DH 19.52% (6 hr) which was 81.12%, while the lowest activity was recorded as 80.49% in PH of DH 13.53% (4 hr) at a concentration of 5 mg/ml. These results were in agreement with reported by Saiga et al. (2003) who found some amino acids such as, histidine, tyrosine, cystine, and tryptophan, especially histidine exhibits strong radical scavenging activity, which were higher in the PH at DH 11.96% than other PH.

The IC_50_ value is applied as an indicator to evaluate the ABTS scavenging capacity. In Figure 6a, the IC_50_ value observed that the PH of DH 19.52% was more active (1.59 mg/ml) than PH of DH 11.96% (1.67 mg/ml), while PH of DH 13.53% was less active (1.76 mg/ml), this might be due to the smaller molecular weight peptides (<1,000 Da) as shown in Figure 2. Stefanović et al. (2014) found that smaller molecular mass peptides (<1000Da) led to a higher ABTS radical scavenging activity.

#### 3.10.2 DPPH radical scavenging activity

DPPH free radical scavenging activities of GTPH are demonstrated in Figure 6b at concentrations of 1–20 mg/ml. The result shows that the highest DPPH scavenging capacity was 75.89% at DH 11.96% followed by 63.35% at DH 13.53%. Lowest value (59.09%) was obtained in PH at DH 19.52% with the concentration of 20 mg/ml, these results may have relationship with some amino acids composition. Our all results were higher than reported by Damgaard et al. (2014). Park et al. (2016) reported that amino acids e.g. threonine, isoleucine, and valine, beside to hydrophobic amino acids strongly contribute to positive effects on DPPH scavenging activities.

The IC_50_ activity of PH is a concentration used for inhibition of DPPH to 50%. In Figure 6b, the IC_50_ value shown that the PH obtained at DH 11.96% was highly active (12.50 mg/ml) followed by PH of DH 13.53% (15.97mg/ml), while PH at DH 19.52% was lowest active (17.58 mg/ml), these finding may be attributed to high amount of hydrophobic amino acids including alanine, valine, leucine, isoleucine, cysteine and phenylalanine in PH of DH 11.96%.

#### 3.10.3 Reducing power

The reducing power was indicated to increase the absorbance are presented in Figure 6c. Reducing power capacity of GTPH at DH 19.52% was slightly higher (0.88) at concentration of 15 mg/ml than DH 11.96% (0.84) and DH 13.53% (0.81) PHs, however, showing nonsignificant difference (*p* < .05). These results possibly due to acidic and free amino acids. Park et al. (2016) reported that acidic amino acids such as aspartic acid and glutamic acid have strong positive effects on reducing power. In addition, Binsi et al. (2016) mentioned to protein hydrolysate peptides of aquatics fish having a molecular weight of < 5000Da were mainly responsible to antioxidant activity. In this study reducing power capabilities were higher than the previous results found by Binsi et al. (2016) of engraved catfish.

#### 3.10.4 Hydroxyl radical scavenging activity

The hydroxyl radical scavenging activities of GTPHs are presented in Figure 6d. The activity of hydroxyl radical scavenging was the highest (95.25%) in PH with DH 11.96% followed by 81.41% in PH of DH 13.53%, while lowest value (75.72%) in PH of DH 19.52% at the same concentration of 15 mg/ml. In the current study, low DH achieved the highest hydroxyl radical scavenging activity. These is results closely related with the result of He et al. (2015) from Anchovy protein hydrolysate. Our results was higher than by Gao et al. (2019).The lower IC_50_ value means the higher free radical scavenging ability. In Figure 6d, IC_50_ value obtained at PH of DH 11.96% was the best active (7.89 mg/ml) than the PH of DH 13.53% and DH 19.52% which amounted 9.97% and 9.98%, respectively, with no significant differences (*p* < .05) between PH of DH 13.53% and DH 19.52%.

#### 3.10.5 Metal chelating properties

##### Fe^2+^ and Cu^2+^ Chelating activity

The metal chelating activity of GTPH was evaluated and expressed as a percentage at different concentration (mg/mL). Iron (II) and copper (II) ion chelating capacities of PHs are presented in Figure 6e and f. The Fe^2+^ chelating activities observed that PH of DH 11.96% was a highly strong metal (Fe^2+^) chelating activity (63.25%) at a concentration of 20 mg/ml, while PH of DH 13.53% and DH 19.52% were 56.17 and 52%, respectively. These activities may be related with acidic and basic amino acids. On the other hand, Cu^2+^ chelating activity is displayed in Figure 6f. PH of DH 11.96% was significantly high 66.90% at the concentration of 6 mg/ml followed by PH of DH 19.52% (65.01%), without significant difference with PH of DH 13.53% which was 64.78%. These results may be due to essential amino acids specially histidine. Torres‐Fuentes et al. (2011) reported that high amount of histidine content, which provided the highly copper chelating activity due to its imidazole ring.

The capability of PH to Fe^2+^ and Cu^2+^ chelating activities were confirmed in terms of their IC_50_ values. In Fe^2+^ chelating, DH 11.96% PH given IC_50_ value by a concentration of 14.19 mg/ml but DH 13.53% and DH 19.52% PH were provided IC_50_ values at a concentration of 17.68 and 18.33 mg/ml, respectively. On the other hand, Cu^2+^ chelating of PH at DH 11.96% was the highly active (3.73 mg/ml) shadowed by PH of DH 13.53% and 19.52%, whereas there were no significant differences (*p* < .05) between PH of DH 13.53% and 19.52% activities. Results of the present study suggest that, the metal chelating properties were a significantly high in GTPH, which can be retard the oxidation reaction of volatile compounds in storage food duration (Saiga et al., 2003).

## 4 CONCLUSIONS

Enzymatic hydrolysis conditions significantly affecting DH, functional properties, and antioxidant activities of GTPH. Protein solubility was related to molecular weights and amino acids profile. The highest value of EAI achieved at pH 6 and the lowest at pH 4, while the ESI was the highest at pH 10. The pH level and DH significantly affecting foaming properties. The best results of WHC and OHC achieved in PH at DH of 13.53% and 19.52%. Lightness index value significantly decreased when the DH increased, while redness and yellowness gradually increased. Among antioxidant activities evaluated, ABTS, DPPH, hydroxyl radical, and metal chelating (Fe^2+^ and Cu^2+^) were significantly higher in PH of DH 11.96%, whereas FRAP activity at DH 19.52%. Therefore, PH obtained from grass turtle muscles has good functional properties and can be used as a natural source of antioxidant peptides, hence could be applied in food and pharmaceuticals industries. Thus, further studies to identification of the specific peptides and amino acid sequences are needed to improve the functional foods.

## CONFLICT OF INTEREST

The authors declare no conflict of interest.

## ETHICAL STATEMENT

This research article does not contain any studies with human participants or animals performed by any of the authors.
